# Immune checkpoint inhibition increases antigen-specific T cell response in head and neck cancer

**DOI:** 10.1038/s41598-026-38740-z

**Published:** 2026-02-09

**Authors:** Patrick J. Schuler, Franziska Oliveri, Lisa Puntigam, Klara Six, Carlotta Kaißer, Julia Maier, Simon Laban, Adrian von Witzleben, Cornelia Brunner, David A. C. Messerer, Hubert Schrezenmeier, Thomas K. Hoffmann, Marlies Goetz, Jochen Greiner

**Affiliations:** 1 Department of Otorhinolaryngology, University Hospital Heidelberg, 69120 Heidelberg, Germany; 2 Department of Otorhinolaryngology, Head and Neck Surgery, University Hospital Ulm, 89075 Ulm, Germany; 3 Institute of Pathology, University Hospital Ulm, 89081 Ulm, Germany; 4 Department of Internal Medicine III, University Hospital Ulm, 89081 Ulm, Germany; 5 Institute for Transfusion Medicine, University Hospital Ulm, 89073 Ulm, Germany; 6 Institute for Clinical Transfusion Medicine and Immunogenetics Ulm, German Red Cross Blood Transfusion Service Baden-Württemberg-Hessen and University Hospital Ulm, 89081 Ulm, Germany; 7 Department of Internal Medicine, Diakonie Hospital Stuttgart, Rosenbergstr. 38, 70176 Stuttgart, Germany

**Keywords:** Anti-LAG-3 (Lymphocyte activation gene 3), Anti-PD-1 (Programmed death-1, aPD-1), Anti-TIM-3 (T cell immunoglobulin and mucin-domain containing-3), Head and neck cancer (HNC), Immune checkpoints, Melanoma-associated antigen (MAGE), New York esophageal cell carcinoma 1 (NY-ESO-1), Preferentially Expressed Antigen in Melanoma (PRAME), Tumour-associated antigens (TAA), Cancer, Immunology, Oncology

## Abstract

Therapeutic strategies which target immune checkpoint markers and enable the immune system to initiate immune responses against tumor cells represent a major advancement in cancer therapy. The response rate to anti-PD-1 checkpoint inhibition in head and neck cancer is about 20%, which underlines the importance of finding further immune-based treatment options. Furthermore, the effects of immune checkpoint inhibitors on antigen-specific T cells have not yet been sufficiently explored, therefore more detailed investigations are required. In mixed lymphocyte-peptide cultures, specific cytotoxic T cells were generated against various tumor-associated antigens. Several tumor-associated antigens such as MAGE, PRAME and NY-ESO-1 were identified as the most potent immunostimulatory agents. The immune response of those specific T cells against head and neck cancer cell lines was measured in ELISPOT assays. The influence of PD-1 and other immune checkpoints on the peptide-specific immune response was investigated with T cells from healthy donors in conjunction with HNSCC tumor cells. Especially the anti-PD-1 antibody is able to increase antigen-specific immune responses. The combination of anti-PD-1 and other checkpoint inhibitors, like LAG-3 or TIM-3, lead to little or no synergistic effects. Antigen-specific vaccination in combination with PD-1 checkpoint inhibition may therefore be a potential future therapeutic option in head and neck cancer to generate an enhanced anti-tumor immune response. Based on the study findings, an increase antigen-specific immune response by vaccinating the patient with a tumor-associated peptide in combination with anti-PD-1 antibody would be advantageous in HNSCC. Efforts into finding and developing new combination therapies should be further advanced.

**Supplementary Information:**

The online version contains supplementary material available at 10.1038/s41598-026-38740-z.

## Introduction

Head and neck cancers are a heterogeneous group of cancers that arise from the squamous epithelium of different locations in the head and neck region. Most head and neck cancers are located in the oral cavity, pharynx and larynx and are referred to as head and neck squamous cell carcinoma (HNSCC). These tumors are mostly associated with tobacco and/or alcohol abuse. Cancers of the oropharynx are increasingly attributed to human papillomavirus (HPV) infection. HNSCC can be therefore differentiated into HPV-negative and HPV-positive cancers. According to the international guidelines, the treatment recommendation is not yet influenced by the HPV-status of the tumor. HNC ranks seventh worldwide, with over 660,000 new cases and approximately 325,000 deaths each year. HNCs are classified by anatomic subsite according to the World Health Organization’s International Classification of Diseases (ICD-10)^1^.

Depending on the location and the patients preference, treatment for early stages of HNSCC consists of surgery or radiotherapy. The treatment approach of locally advanced HNSCC is usually multifaceted and consists of surgery followed by radio(chemo)therapy, or definitive radiochemotherapy^2^. More than 50% of the patients relapse within 3 years. Patients, who experience cancer progression within 3 months after platinum-based chemotherapy of primary or recurrent disease, have a median survival rate of 6 months or less^3^.

HNSCC require a highly versatile and flexible treatment methodology to ensure therapeutic success. Therefore, new immunological treatment methods are urgently needed to better control the disease in the first instance and, in the best case scenario, keep it in complete remission in the long term^2^. Our research examines how immune checkpoint inhibitors affect antigen recognition by specially activated T cells and their stimulation and cytotoxic function through Interferon-γ and granzyme B measurement. This work lays groundwork for a clinical trial exploring combined peptide vaccines and immune checkpoint inhibition in HNSCC.

Recent advances in immunotherapy have led to the establishment of anti-PD-1 immune checkpoint therapies with Nivolumab or Pembrolizumab which are used for cisplatin-refractory or metastatic HNSCC. Moreover, the FDA has approved the immune checkpoints Pembrolizumab and Nivolumab for the treatment of recurrent or metastatic HNSCC and Pembrolizumab as first-line therapy for unresectable disease^3–5^. The response rate for anti-PD-1 in HNSCC is around 20%, highlighting the importance to find more immune-based treatment options^3^.

Evasion of the immune system is a major mechanism that can lead to uncontrolled proliferation and metastasis of tumor cells^6,7^. Natural control mechanisms such as immune checkpoints are bypassed by the tumor and weaken the anti-tumor immune response. Blockade of these inhibitory signaling pathways with immune checkpoints is already showing positive effects in cancer therapy^8,9^.

Immunotherapy for malignant diseases includes a variety of therapeutic options. Oftentimes, different therapeutic options are combined in oder to improve response rates of patients particularly because they are often directed against different target structures. In this study, we could identify T cell-specific immune responses against several tumor-associated antigens (TAA) in peripheral blood mononuclear cells (PBMCs) from healthy donors. The number of specific T cells vary in the samples of healthy donors, since a certain frequency of T cells must be present in the sample, because only donors with active T cells can elicit specific responses.

TAA are mainly, but not exclusively, expressed by tumor cells. These immunogenic target structures are recognized by CD8 + T cells with the support of CD4 + T helper cells, enabling CD8 + cells to subsequently eradicate tumor cells. Some of them additionally trigger a humoral immune response. The Melanoma-associated antigen (MAGE), New York esophageal cell carcinoma 1 (NY-ESO-1) and Preferentially Expressed Antigen in Melanoma (PRAME), which we selected, are relevant antigens as they are among the most frequently expressed TAA in HNSCC^10–13^. TAA-specific T cells served as the basis for investigating the effect of immune checkpoints on specific immune responses and for examining whether these effects can be enhanced by using different immune checkpoints alone and in combination. The effects of immune checkpoint combinations on antigen-specific T cells have not yet been sufficiently investigated ex vivo in HNSCC and are therefore examined in this study. To our knowledge, most of the ex vivo studies conducted to date have focused mainly on anti-PD-1 monotherapies and combinations with other checkpoint inhibitors have only been studied to a limited extent, and if so, only with anti-CTLA-4^14,15^.

In HNSCC, the main immune checkpoint pathways involved in suppressing the immune system are PD-1/PD-L1, cytotoxic T lymphocyte antigen 4 (CTLA-4), T cell immunoglobulin mucin 3 (TIM-3), lymphocyte activation gene 3 (LAG-3), and T cell immunoglobulin and immunoreceptor tyrosine-based inhibitory motif (TIGIT)^16^. The immunotherapeutics used in this study were anti-PD-1 (Nivolumab), anti-TIM-3 (Cobolimab) and anti-LAG-3 (A249221) alone and in combination.

Our research focuses on expanding knowledge on the use of various immune checkpoints in the treatment of HNSCC in combination with vaccination with tumor-associated peptides. Our goal is to uncover additional treatment options with immune checkpoints and their potential combinations with other therapeutic agents, such as TAA as a vaccine to specifically activate T cells. These innovative approaches of vaccines in combination with checkpoint inhibitors are being explored to increase treatment efficacy and improve treatment outcomes for patients in this challenging area of oncology.

## Methods

### Sample preparation, storage and cell culture

Peripheral blood mononuclear cells (PBMC) from healthy donors (HD) were separated by Ficoll (Pan Biotech, Aidenbach, Germany) density gradient centrifugation, cryopreserved and stored in liquid nitrogen. HD samples were obtained from the Blood Donor Service of the German Red Cross Ulm. The tumor cell line UT-SCC-33 (University of Turku-Squamous Cell Carcinoma-33, HPV negative, RRID: CVCL_7837) was cultured in T175 flasks with MEM α cell culture medium with 10% FCS and 1% Cell shield at 37 °C, 5% CO_2_ in waterlogged air. Cells were split according to confluency, which corresponded to a split of 1:4 or 1:5 twice a week and used pulsed or not pulsed with TAA as antigen presenting cells (APC) in the ELISPOT.

### Immunohistochemistry

In immunohistochemistry UT-SCC-33 were negative for MAGE and PRAME, however moderately positive for NY-ESO-1 in the nucleus and cytoplasm.

### Mixed lymphocyte peptide culture (MLPC)

In MLPC, whole PBMCs of healthy donors were used for the experiments without any separation steps. Antigen-specific CD8 + allogeneic T cells were produced from whole PBMC HD samples, yielding effector cells for subsequent analyses. Briefly, whole PBMC samples were thawed, counted and divided in two fractions. One fraction, employed as APC, was irradiated with 30 Gray and pulsed with the respective peptides for 2 h at 37 °C. Thereafter, the pulsed APC fraction was mixed with the second PBMCs fraction as effector cells at a ratio of 1:1. On day two IL-2 (2.5 ng/ml) and IL-7 (20 ng/ml) were added. After seven to nine days of incubation, cells were used for functional assays with or without the addition of checkpoint inhibitors.

### Viral- and tumor-associated antigens

Cytomegalovirus (CMV, NLVPMVATV) or Influenza Matrix Protein (IMP, GILGFVFTL) derived peptides served as positive controls, according to results in the pretests for each HD. No peptide (NoP) served as a negative control. In an initial analysis (Figure S1) we tested five TAA (Renal cell carcinoma-associated antigen (G250, QLL LSL LLL); Melanoma-associated antigen (MAGE, SLLMWITQC^13^); New York esophageal squamous cell carcinoma-1 (NY-ESO-1, SLLMWITQC); Preferentially Expressed Antigen in Melanoma (PRAME, ALYVDSLFFL), and Receptor for hyaluronic acid-mediated motility (RHAMM, ILSLELMK)) to determine which TAA should be selected for further testing ± immune checkpoints. MAGE, NY-ESO-1 and PRAME, the peptides with the most robust immune responses, were chosen for further analysis (Figure S1A, B). All peptides were HLA-A2 restricted and were used at a concentration of 20 µg/ml, other concentrations of peptides were tested (Figure S1C).

### Addition of Nivolumab to cell culture

In line with the results of titrations^17^, 5 µg/ml of the immune checkpoints were added to the stimulated T cells from MPLC and peptide-pulsed HNSCC cell line UTSCC-33 for 24 h in the ELISPOT wells at 37 °C, 5% CO_2_ in waterlogged air. In this way, the direct effect of immune checkpoints on CD8 + T cells with the support from CD4 + T helper cells was measured. 24 h was chosen because multiple time points were examined and 24 h yielded the best results (Figure S2).

### ELISPOT (enzyme-linked-immuno-spot)

IFNγ (Mabtech, Nacka Strand, Sweden) and granzyme B (GrB, BD, Heidelberg, Germany). ELISPOTs were performed according to the manufacturer’s instructions. Briefly, Membrane bottom 96 well plates were coated with a solid antibody phase against IFNγ or GrB over night at 4 °C. Subsequently, the blocked membranes were incubated with stimulated T cells from MLPC and/or the cell line UT-SCC-33 as APC, pulsed with or without peptide in the presence of 2.5 µg/ml β2-microglobulin at a ratio of 5:1. The relevant immune checkpoints were introduced alone or combined into the T cell/cancer cell line co-culture. The antigen-specific T cells with or without UT-SCC-33 ± TAA, ± immune checkpoints and UT-SCC-33 alone, were applied in triplicate to the ELISPOT plate and incubated for 24 h. The cytokines which bound to the solid antibody phase were visualized by specific, biotin-coupled antibodies, alkaline phosphatase and by the appropriate substrate. The evaluation was carried out by using an ELISPOT reader. Figure S3 Controls and results with/without addition of IL-2/IL-7.

### Flow cytometry

The frequency of TAA-specific CD8 + T lymphocytes was determined after 7–9 days of MLPC by staining with an anti-CD8 antibody and HLA-A2/MAGE or /NY-ESO-1 pentamer (Proimmune, Oxford, UK). The specific T cells were stained with the respective pentamer at 1 µg per test and incubated for 40 min at room temperature in the dark. Antibodies directed against CD8 (FITC) and CD4 (PerCP) (BD, Heidelberg, Germany) were then added and incubated for 20 min at 4 °C in the dark. After a single wash with PBS, the stained cells were fixed with 1% formaldehyde (Sigma Aldrich) and then analyzed by flow cytometry. Whenever possible, at least 100.000 events were collected for analysis. Each sample was run with an appropriate isotype and Fluorescence minus one (FMO) control to define the gate of positive cells. Analysis was performed on FSC/SCC-driven lymphocytes to exclude dead cells and debris and on CD8 + /CD4 − T lymphocytes to assess responses to the respective peptide. Ligand detection was performed according to manufacturer’s instruction either extracellularly or intracellularly. Phosphatidylserine was analyzed extracellularly (detected by Annexin V staining). All multicolor panels were compensated with single stained samples on the BD FACSAria™ III. FMO controls were used to determine cutoffs for positivity. Non-specific antibody binding was excluded by setting up isotype controls. Antibodies used for ligand detection: CD8 PECy7/CD279 (FITC/PerCP, PD-1)/CD274 (PE, PD-L1)/CD273 (APC, PD-L2), (all Biolegend), LAG-3 (CD223, FITC, BD), HLA-DR (PE, BD), Galectin-3 (AlexaFlour647, BD), TIM-3 (CD366, APC, Biolegend), Galectin-9 (FITC, Biolegend), Annexin V (FITC, Biolegend). The Cytec lymphocyte checkpoint panel from the Core Facility Immune Monitoring at the University of Ulm was used to detect the co-stimulatory molecules.

### Statistical analysis

Statistical tests were performed using GraphPad PRISM v8. The program was also used to evaluate assays, for comprehensive analysis, for organizing data and for generating graphs. As a statistical analysis we used the RM one-way Anova test. * = *p* < 0.05, ** = *p* < 0.01, *** = *p* < 0.001, **** = *p* < 0.0001.

### Sharing statement

For original data, please contact marlies.goetz@alumni.uni-ulm.de.

## Results

**Fig. 1:**
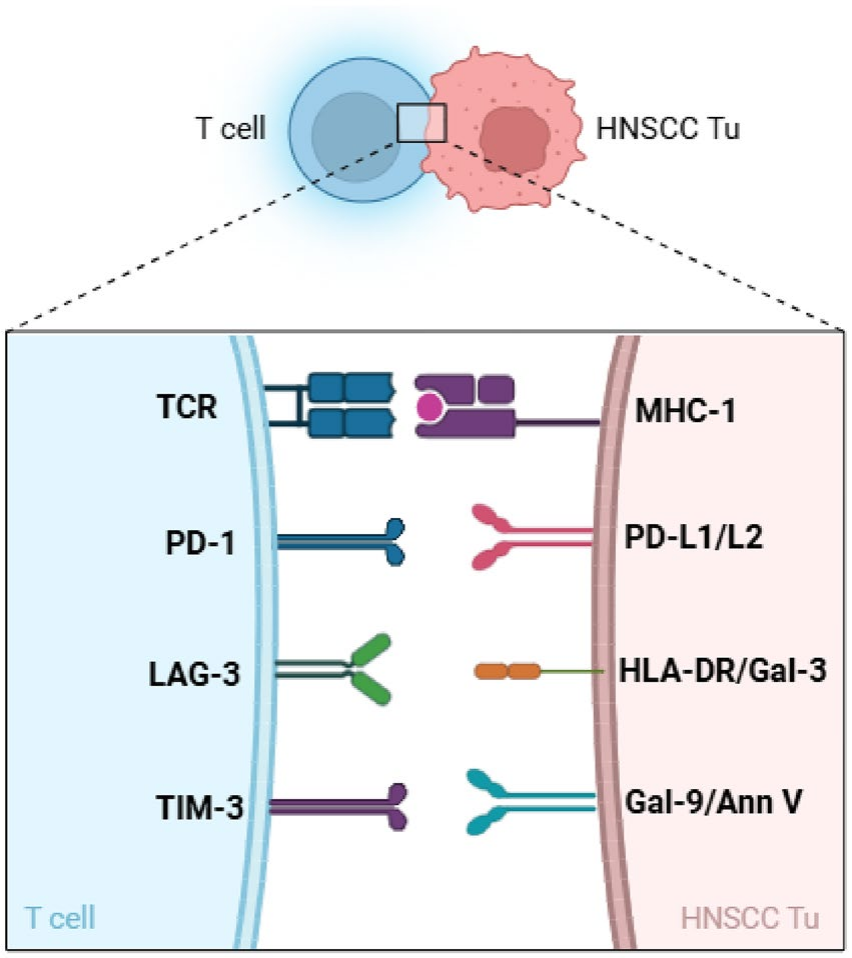
Graphical abstract. Relevance of immune checkpoint molecules for T cell specific antigen recognition in HNSCC tumors (created by bio Render).

In view of the relatively poor response of Head and Neck Squamous Cell Carcinoma (HNSCC) patients to immune checkpoint therapy, our research focused on investigating PD-1 blockade combined with other immune checkpoints in the context of tumor antigen-specific immune responses against HNSCC tumor cells (Fig. 1). In preliminary test five potentially immunogenic peptides (G250, MAGE, NY-ESO-1 PRAME and RHAMM) were examined, with MAGE, NY-ESO-1, and PRAME eliciting the strongest immune responses^18^ (Figure S1A,B). These TAA also showed robust immune responses in other tumor entities^9,19,20^.

**Fig. 2:**
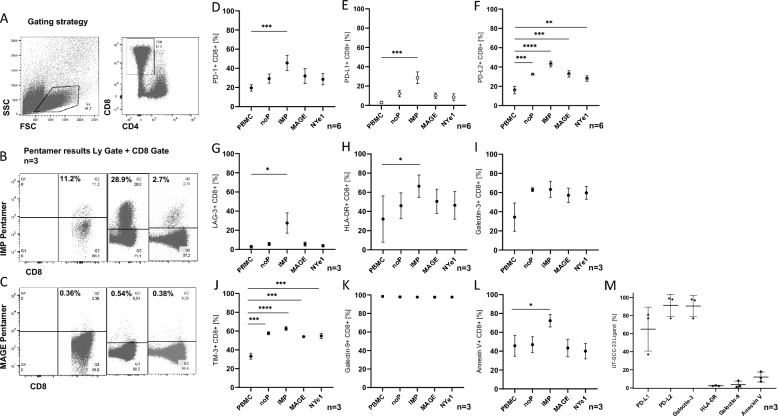
Flow cytometry. (**A**–**C**) PBMC or T cell alone or T cell/cell line co-culture from ELISPOT for Pentamer expression. (**A**) Gating strategy. (**B**) In the SSC/FSC co-culture view, we selected a lymphocyte gate (Ly 47.4%) and then selected to display CD4 against CD8 cells to choose the gate for CD8 + /CD4- cells (CD8 47.2%) to then visualize the CD8 + /IMP pentamer + cells. The IMP pentamer served as positive control, n = 3HD (three different HD). (**C**) Selection of gate was as above to then visualize the CD8 + /MAGE pentamer + cells. The MAGE pentamer showed a lower expression than the positive control, n = 3HD. (**D**–**L**). Expression of immune checkpoints and their ligands on CD8 + cells. Lymphocytes were analyzed from untreated PBMCs compared to pre-stimulated MLPC cells co-cultured with UT-SCC-33 presenting the respective peptide or no Peptide (NoP). (**F**) The differences in PD-L2 expression were significant when comparing PBMC with cells from MLPC (PBMC vs. NoP p 0.0003, PBMC vs. IMP *p* < 0.0001, PBMC vs. MAGE p 0.0002, PBMC vs. NY-ESO-1 p 0.0064). (**G**–**I**) Expression of LAG-3 and its ligands HLA-DR and Galectin-3. As for LAG-3 expression a significant increase from PBMC to IMP was seen. The HLA-DR ligand showed a similar pattern. Galectin-3 expression differences were not significant. (**J-L**) Expression of TIM-3 and its ligands. Significant differences were seen for TIM-3 extracellular staining (PBMC vs. NoP p 0.0003, PBMC vs. IMP *p* < 0.0001, PBMC vs. MAGE p 0.0009, PBMC vs. NY-ESO-1 p 0.0007). Gal-9 expression was high for all samples. For phosphatidylserine significant expression differences were detected between PBMC with IMP. (**M**) Expression of Ligands on UT-SCC-33 (n = 3). Flow cytometric expression of PD-1, LAG-3 and TIM-3 ligands. Data are depicted as mean ± SD.

Our focus was initially on FACS analysis of T cells, with an emphasis on pentamer expression, in order to demonstrate that specific stimulation with TAA is effective. (Fig. 2A) Gating strategy SSC/FSC T cell gate and CD8 + /CD4 − cells. (Fig. 2B) The IMP pentamer was used as positive control. Mean CD8 + /IMP pentamer + cells were 14.3%, n = 3 HD (different HD measured). (Fig. 2C) The MAGE pentamer as TAA showed, as expected, lower expression of CD8 + /MAGE pentamer + cells of a mean of 0.43%, n = 3 HD. (Fig. 2D–M) detection of the immune checkpoints as well of their ligands on the PBMC compared to CD8 + T cells and on the cell line UT-SCC-33. The expression of both immune checkpoints and their ligands is crucial, as inhibition of binding to immune checkpoints can only occur if both are present. (D–F) Flow cytometric expression profile of PD-1 ligands: PD-1 mean values of CD8 + /ligand in % were for all groups 29% and PD-L1 mean all groups: 16% and PD-L2 mean all groups: 33%; n = 6 HD. (G–I) Flow cytometric expression profile of LAG-3 and its ligands: mean values of CD8 + for all groups measured: LAG-3 9%, HLA-DR 49%, Gal-3 55%; n = 3 HD. (J–l) Flow cytometric expression profile of TIM-3 and its ligands. Mean values for CD8 + in %: TIM-3 53%, Gal-9 98%, AnnexinV 50%. (M) flow cytometric expression profile for each relevant ligand of the cell line UT-SCC-33/ligand, mean values: PD-L1 65%, PD-L2 91%, Galectin-3 91%, HLA-DR 2%, Galectin-9 4%, Annexin V 12%; n = 6 (UT-SCC-33 from different passages). In some cases, there are significant differences in expression between the various stimulated T cells and PBMCs. Most ligands are expressed quite well by T cells, whereas the UT-SCC-33 cell line does not express all ligands, but expresses one ligand each of LAG-3 and TIM-3.

In the course of our investigations, we have set up a test system to screen the various immunotherapeutic effects. To determine the immune responses, whole PBMCs without any separation procedures from HD were used, which were stimulated with TAA in an MLPC for 7–9 days and then treated with immune checkpoint inhibitors for 24 h. Readout was performed using ELISPOT and various FACS analyses. We observed TAA reaction in PBMCs from HD. This shows that these specific T cells are also present to a certain extent in HD and that they can be activated and expanded in vitro.

**Fig. 3:**
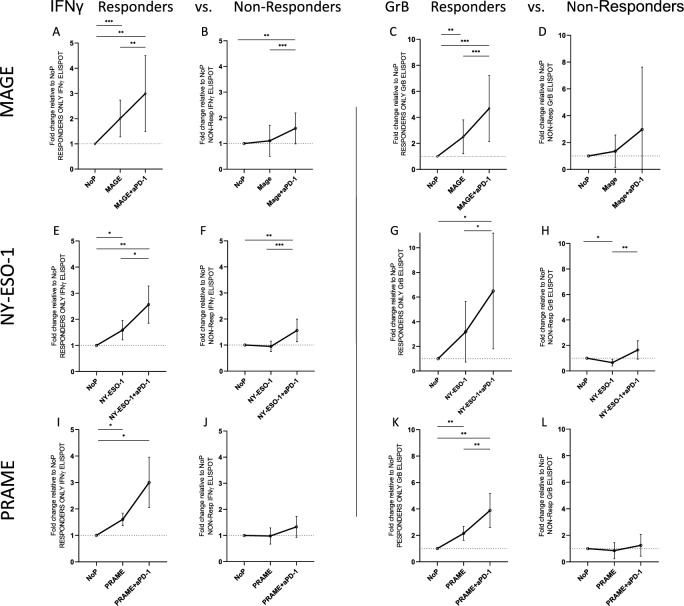
IFNγ and GrB secretion in ELISPOT assays. PBMC derived from healthy donors (HD) were stimulated with the different TAA in MLPC. The fold change is shown in relation to the untreated immune cells (no Peptide, NoP). Each row first shows IFNγ secretion by responders (R) compared to non-responders (NR) and then the respective GrB secretion R vs. NR. Specific immune responses are shown after co-incubation with HNSCC tumor cells. (**A**) in R immune response increased significantly when comparing NoP with MAGE (p 0.0009), and comparing MAGE with MAGE/aPD-1 (p 0.0012) and NoP with MAGE/aPD-1 (p 0.0061). (**B**) non-responders only by MAGE and MAGE/anti-PD-1 stimulation (p 0.0003) and NoP with MAGE/aPD-1 (p 0.0013; 13R vs. 19NR). (**C**) GrB secretion R (NoP vs. MAGE p 0.0023; NoP vs. MAGE/aPD-1 p 0.0003; MAGE vs. MAGE/aPD-1 p 0.0008) (**D**) compared to NR. (**E**–**H**) NY-ESO-1 stimulation IFNγ R vs. NR (6R/11NR; R: NoP vs. NY-ESO-1 p 0.0266; NoP vs. NY-ESO-1/ aPD-1 p 0.0070; NY-ESO-1 vs NY-ESO-1/ aPD-1 p 0.0108) and GrB R vs. NR. (**I**, **J**) PRAME stimulation IFNγ R vs. NR (5R/10NR) and (**K, L**) GrB R vs. NR. Data is shown as mean ± SD. Statistical significance was determined using one-way ANOVA. * = *p* < 0.05, ** = *p* < 0.01, *** = *p* < 0.001.

**Fig. 4:**
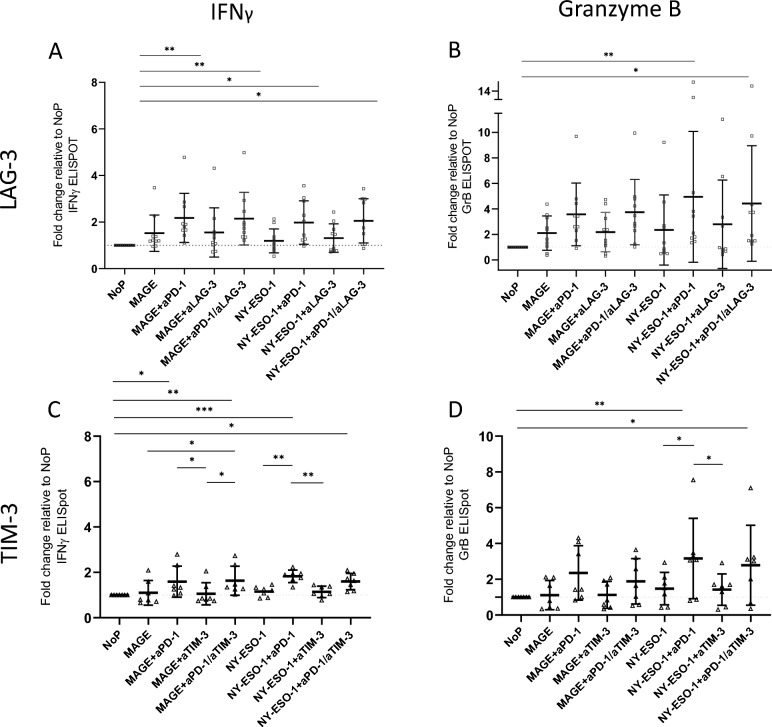
IFNγ and GrB secretion in ELISPOT assays. Stimulation with LAG-3 and TIM-3 ± aPD-1 (n = 10 HD). (**A**) MAGE and NY-ESO-1 ± aPD-1 ± LAG-3 IFNγ (NoP vs. MAGE/aPD-1 p 0.0043; NoP vs. MAGE/aPD-1/aLAG-3 p 0.0062; NoP vs. NY-ESO-1/aPD-1 p 0.0338; NoP vs. NY-ESO-1/aPD-1/aLAG-3 p 0.0164). (**B**) MAGE and NY-ESO-1 ± aPD-1 ± LAG-3 GrB (NoP vs. NY-ESO-1/aPD-1 p 0.0044; NoP vs. NY-ESO-1/aPD-1/aLAG-3 p 0.0226). (**C**) MAGE and NY-ESO-1 ± aPD-1 ± TIM-3 IFNγ (NoP vs. MAGE/ aPD-1 p 0.0130; NoP vs. MAGE/aPD-1/aTIM-3 p 0.0001; NoP vs. NY-ESO-1/aPD-1/aTIM-3 p 0.0127; MAGE vs. MAGE/aPD-1/aTIM-3 p 0.0368; MAGE/aPD-1 vs. MAGE/aTIM-3 p 0.0351; MAGE/aTIM-3 vs. MAGE/aPD-1/aTIM-3 p 0.0181; NY-ESO-1 vs. NY-ESO-1/aPD-1 p 0.0030; NY-ESO-1/aPD-1 vs. NY-ESO-1/ aTIM-3 p 0.0023). (**D**) MAGE and NY-ESO-1 ± aPD-1 ± TIM-3 GrB. The fold change is shown in relation to no Peptide (NoP) (NoP vs. NY-ESO-1/aPD-1 p 0.0012; NoP vs. NY-ESO-1/aPD-1/aTIM-3 p 0.0132; NY-ESO-1 vs. NY-ESO-1/aPD-1 p 0.0231; NY-ESO-1/aPD-1 vs. NY-ESO-1/ aTIM-3 p 0.0170). Statistical significance was determined using one-way ANOVA. * = *p* < 0.05, ** = *p* < 0.01, *** = *p* < 0.001.

**Fig. 5:**
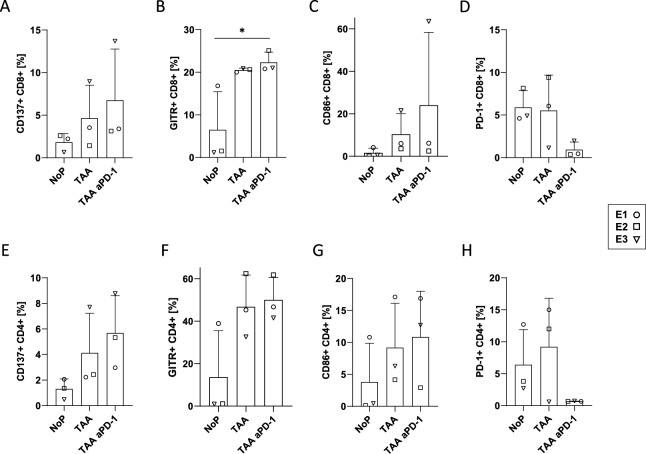
Co-stimulatory molecules. (**A**–**D**) Expression of co-stimulatory molecules and checkpoints on CD8 + cells. (**E**–**H**) Expression of co-stimulatory molecules and checkpoints on CD4 + cells. In each case comparing cells with different stimulations: NoP/TAA/TAA + aPD-1. We see a tendency of an increase of co-stimulatory molecules, when we compare NoP (no stimulation) with cells stimulated with a TAA and all the more so with anti-PD-1. (**D**, **H**) When the immune checkpoint PD-1 is analyzed, the opposite effect is observed.

In ELISPOT assays antigen specific T cells co-cultured with the cell line UT-SCC-33 were used ± TAA ± immune checkpoints alone or in combination. The fold change is shown in relation to the untreated immune cells (NoP) co-cultured with unstimulated UT-SCC-33 as APC, each with the addition of β2-microglobulin only. * = *p* < 0.05, ** = *p* < 0.01, *** = *p* < 0.001 (Figs. 3, 4 and 5).

We could demonstrate a robust immune response by addition of TAA compared to the NoP control. The TAA response was enhanced by the addition of the checkpoint inhibitor anti-PD-1 always in conjunction with the TAA pulsed cell line UT-SCC-33. Of note, NoP T cells without addition of a cell line could almost not be stimulated with anti-PD-1, and we observed no reactions when no IL-2/IL-7 was added to the MLPC (Figure S3A–C).

Figure 3 first shows results of responders (R) and subsequently of the non-responders (NR), whereby an increase of 25% from NoP to the respective TAA was classified as R. The same T cells are also shown for TAA and anti-PD-1. Strong immune responses could be elucidated with the addition of PD-1 to specifically stimulated T cells and co-culture with HNSCC tumor cells.

IFNγ ELISPOT results with a median fold change with the addition of the TAA MAGE in R was 2.0-fold vs. MAGE/anti-PD-1 3.0-fold. In NR for MAGE the increase was 1.1-fold vs. MAGE/anti-PD-1 1.6-fold (Fig. 3A,B). In the GrB ELISPOT we saw an increase in immune responses for MAGE vs. MAGE/anti-PD-1 for the R of 2.5/4.7-fold, representing an increase of more than 80%. When considering the NR, fold change was 1.4 by MAGE stimulation and by MAGE and anti-PD-1 3.1 (Fig. 3C,D). 17 HD were tested for NY-ESO-1. The increase of NY-ESO-1 to NY-ESO-1/anti-PD-1 in R was significant, with an increase of > 60% in IFNγ and > 100% in GrB, the NR, as expected, were low (Fig. 3E–H). As for PRAME, there was a significant increase of immune response when comparing PRAME to PRAME/anti-PD-1 in the group of responders (Fig. 3I,J). In the GrB analyses similar results were seen (Fig. 3K,L). In Fig. 3 a strong immune response by stimulation with the TAA MAGE and PRAME was detected and the immune response increased strongly, with an increase of 60–100%, when anti-PD-1 was added to the TAA pre-stimulated cells of whole PBMC.

In Fig. 4A–D further immune checkpoints were added to our test system to investigate potential alternatives to anti-PD-1, but also possible synergistic effects when used in combination. Results of HD in coculture with a HNSCC cell line are presented, with no distinction between R/NR. The IFNγ/GrB-ELISPOT results show in principle that LAG-3 has no effect either alone or in combination with anti-PD-1. The same applies to TIM-3, which does not elicit an immune response either alone or in combination with anti-PD-1. Our research has shown that combining checkpoints in this context does not provide any additional benefits, suggesting that the use of PD-1 should be continued and a deeper understanding of the basic process gained.

 Expression of co-stimulatory molecules and checkpoints and on CD8 + T cells (Fig. 5A–D). Expression of co-stimulatory molecules and checkpoints and on CD4 + cells. (Fig. 5E–H) Comparison of cells from MLPC, performed with whole PBMC, with different stimulations: NoP/TAA/TAA + anti-PD-1. (A, E) CD8/CD4 expression of the co-stimulatory immune checkpoint molecule CD137; (B, F) expression of the co-stimulatory immune checkpoint molecule GITR; (C, G) of the co-stimulatory molecule CD86 and (D, H) expression of PD-1 on CD8 + and CD4 + cells. There might be a tendency of an increase of co-stimulatory molecules, when we compare NoP (no stimulation) with cells stimulated with the respective TAA and in most groups when anti-PD-1 is added. (D, H) In the PD-1 analysis, by addition of anti-PD-1, the PD-1 site is blocked, which shows that the checkpoint inhibition is successful.

Supplementary Figure S1A,B expression of five TAA used in preliminary tests, to determine which TAA to use for further testing. Figure S1C TAA at a lower concentration of peptides, 1 µg, 2 ug, as well as 10 and 20 µg, 2 µg with and without checkpoints. Figure S2 the fold change in relation to the untreated immune cells co-cultured with the cell line UT-SCC-33 (U33) as APC (No Peptide, NoP). CTL only in each case showed a lower immune stimulation then noP. Figure S2 depicts analyses of the addition of immune checkpoints to MLPC at different time points. Figure S3A, B ELISPOT controls, the positive controls CMV or IMP as well as CTL only ± TAA, ± immune checkpoints and UT-SCC-33 only ± TAA, ± immune checkpoints. Figure S3C results by no addition compared to addition of IL-2/IL-7.

## Discussion

While the effectiveness of immunotherapy in metastatic disease is now recognized, its value in the first-line treatment of locally advanced HNSCC remains controversial and is still being actively investigated^21^. In recurrent and/or metastatic disease, immunotherapy is strategically employed to enhance the efficacy of other conventional therapies, which include palliative chemotherapy^22^. Integrating immunotherapy into existing treatment regimens, may improve treatment outcomes for patients. However, this integration could also introduce new toxicities, necessitate adjustments in dosing regimens, and require innovative clinical trial designs to ensure safety and effectiveness^2^.

In this work, we focus on the question of how different immunotherapies can be optimally combined and coordinated with conventional therapies to improve the existing treatment regimen for HNSCC. Specifically, we examine therapeutic vaccination with a TAA alongside one or more immune checkpoints in vitro. The goal is to identify the most effective combinations that can enhance the immune response while minimizing potential side effects^18,23^.

There are certainly significant limitations to ex vivo studies, such as the absence of a complete tumor microenvironment and systemic and intrinsic influences, such as metabolic and vascular factors. In addition, there are many translational challenges for clinical application, such as the need to find a balance between dose and toxicity, as well as heterogeneity between patients and pharmacokinetic variability, and finally the establishment of validated biomarkers to demonstrate lasting, safe, and clinically meaningful benefits. Nevertheless, ex vivo studies are of some value, as they can provide an indication of the direction to take^24^.

Of the five TAAs tested, three (MAGE, NY-ESO-1 and PRAME) showed a higher response rate and were selected for further investigation^18,25^. TAA are significant for generating robust immune responses, essential for the effectiveness of immunotherapy. The objective of targeted cancer vaccines is to prime antigen-specific T cells, which are indispensable for an effective immune response against cancer^26^. This targeted approach may lead to improved patient outcomes and a better understanding of how to effectively utilize immunotherapy in conjunction with traditional treatment methods.

It is important to emphasize that, to our knowledge, most of the ex vivo examinations conducted to date have focused primarily on anti-PD-1 monotherapies^27^. These studies have provided valuable insights into the efficacy of anti-PD-1 treatments, but they have largely ignored the potential benefits of combining this therapy with other checkpoint inhibitors. Combinations with other checkpoint inhibitors have been investigated only to a limited extent, and when combinations have been tested, it has mostly been with anti-CTLA-4^14^. This limited research raises questions about the full potential of immunotherapy in the treatment of HNSCC^14^.

In our study, we saw that especially the anti-PD-1 antibody is able to increase antigen-specific immune responses. In addition to anti-PD-1, we chose two more immune checkpoints that are particularly relevant in the context of HNSCC^27^. LAG-3 is a transmembrane CD4-related protein that is expressed on the surface of various immune cells, including B cells, dendritic cells (DC), natural killer (NK) cells, and activated T cells. Notably, regulatory T cells (Tregs) express high amounts of LAG-3, which amplifies their immunosuppressive activity by enhancing the production of interleukin-10 (IL-10). The role of LAG-3 in HNSCC has been extensively studied, revealing its significant impact on the immune response. In mouse models, LAG-3 expression increases on T cells and Tregs, indicating its involvement in the tumor microenvironment. Furthermore, anti-LAG-3 antibody treatment has been shown to suppress tumor growth and boost CD8 + T cell responses, highlighting its potential as a therapeutic target^16,28^. Another important immune checkpoint is TIM-3, which is a negative co-stimulatory molecule expressed on CD4 + Th1 cells, CD8 + T cells, Tregs, and various tumor cells. One of the ligands associated with TIM-3 that plays a crucial role in immune regulation is Galectin-9. This ligand promotes apoptosis of Th1 cells, suppresses the activity of CD8 + T cells, and induces the expansion of myeloid-derived suppressor cells (MDSCs), which can further inhibit anti-tumor immunity^16,29^.

An important issue in this context is why only 20% of patients respond to anti-PD-1 therapy, despite its apparent effectiveness in our in vitro analyses. Several factors may contribute to this discrepancy. One possible aspect is the PD-L1 content in the tumor compared to a cell line that consistently expresses the ligand in the same manner^30^. Moreover, in our study we are using T cells from healthy donors who have an intact immune system^31^. This is in stark contrast to late-stage patients, whose immune systems are likely already exhausted due to the disease and previous treatments. Many of these patients have also undergone chemotherapy, which can further weaken the immune system. Therefore, our hypothesis is that earlier use of anti-PD-1 therapy during the course of the disease might be more successful and beneficial for the patient. By administering anti-PD-1 therapy earlier in the treatment process, we may be able to harness a more robust immune response, ultimately leading to better patient outcomes. Therefore, much more effort should be made to investigate the patterns, mechanisms and mode of action of PD-1/PD-L1 in HNSCC^5,30,32,33^.

The current study situation can be summarized as follows. Possible combinations of immunotherapeutics are being tried at times with or without chemotherapy. For example, NCT04470024 tries Multivalent Autophagosome Vaccine, with Anti-PD-1, With or Without GITR Agonist in recurrent or metastatic HNSCC. Whereas NCT04811027 evaluates the safety and efficacy of a soluble LAG-3 Fusion Protein in combination with anti PD-1 against anti PD-1 alone in 1st line metastatic or recurrent HNSCC with PD-L1 positive tumors, and determines the efficacy and safety in patients with PD-L1 negative tumors.

As part of our research, we have developed a test system that can be used to investigate various immunotherapeutics alone and in combination. Healthy CD8 + T cells are used to test the early use of immune checkpoints in combination with TAA vaccination. This test system is currently being used to examine cells from HNSCC patients at different stages of the disease in terms of their response to stimulation with TAA and their subsequent response to immune checkpoints. So far, we have not seen favorable outcomes by combination of multiple checkpoint inhibitors and antigen-specific T cells, and little or no synergistic effect were seen. Nevertheless, we believe it was important to report these findings. Our studies suggest that the use of PD-1 should be continued in combination with vaccine-based stimulation of specific T cells and a deeper understanding of this form of treatment should be gained.

Our study showed that especially the anti-PD-1 antibody is capable of enhancing antigen-specific immune responses ex vivo utilizing healthy donor T cells stimulated with TAA in combination with HNSCC tumor cells. The findings to date suggest that peptide vaccination in combination with immune checkpoint inhibitors such as Nivolumab could be a potential treatment option for HNSCC. Another crucial factor to consider is the timing of when to use anti-PD-1 and whether immune checkpoint inhibitors should be introduced earlier in the disease progression to maximize its effectiveness. Our recommendation based on the study findings is to increase antigen-specific immune responses by vaccinating the patient with a TAA in combination with the anti-PD-1 antibody. This approach should be further developed and tested in a clinical trial. Research and development of new combination therapies need to be further advanced.

## Supplementary Information

Supplementary Information.

## Data Availability

For original data, please contact marlies.goetz@alumni.uni-ulm.de.
