# Supplementary material for: Immune checkpoint inhibition increases antigen-specific T cell response in head and neck cancer
Source: Sci Rep. 2026 Feb 9;16:5583. doi: 10.1038/s41598-026-38740-z (PMC12891685; doi:10.1038/s41598-026-38740-z)
Supplement: Supplementary file 1 — Supplementary Information. [file 41598_2026_38740_MOESM1_ESM.pdf]

Supplementary Figure S1

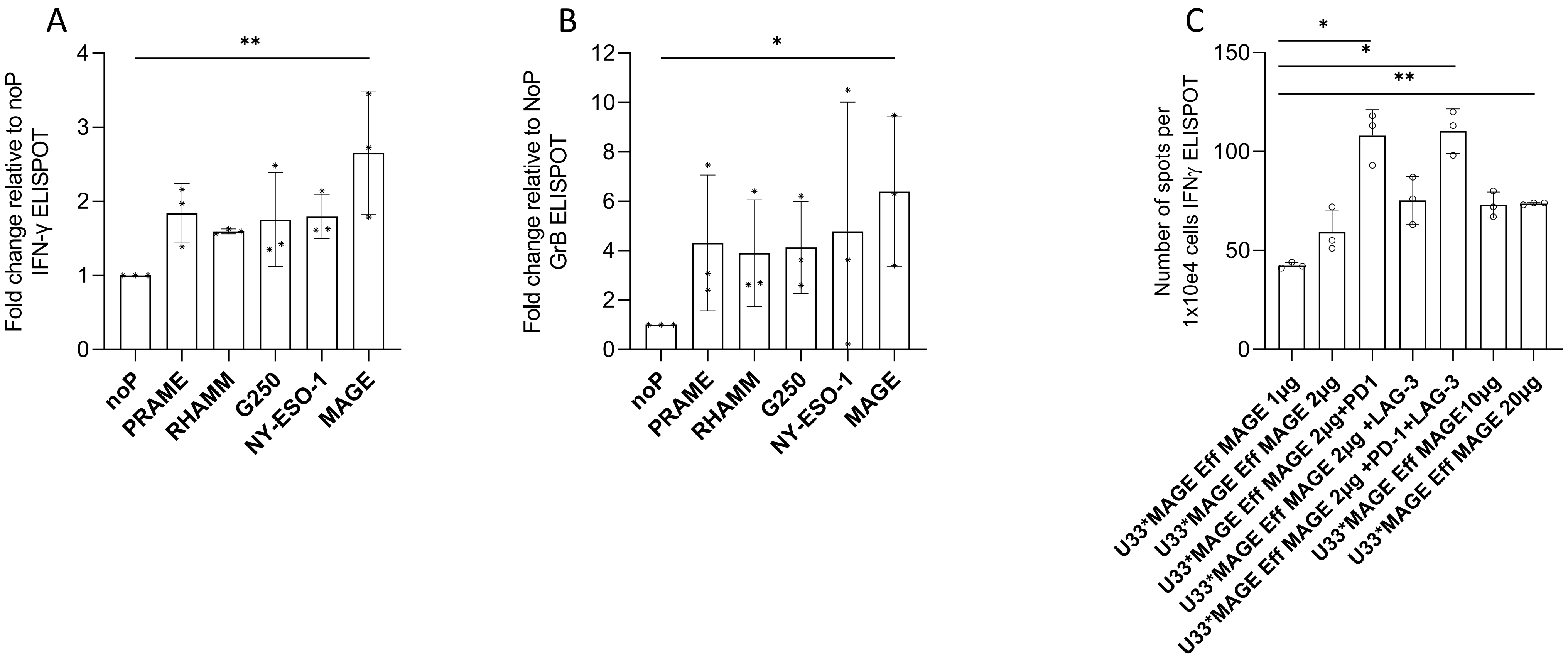

Figure S1 (A, B) expression of five TAA used in preliminary experiments.

Figure S1 (C) TAA at a lower concentration of peptides 1, 2 ug, as well as 10 and 20μg, 2μg with and without checkpoints.

Supplementary Figure S2

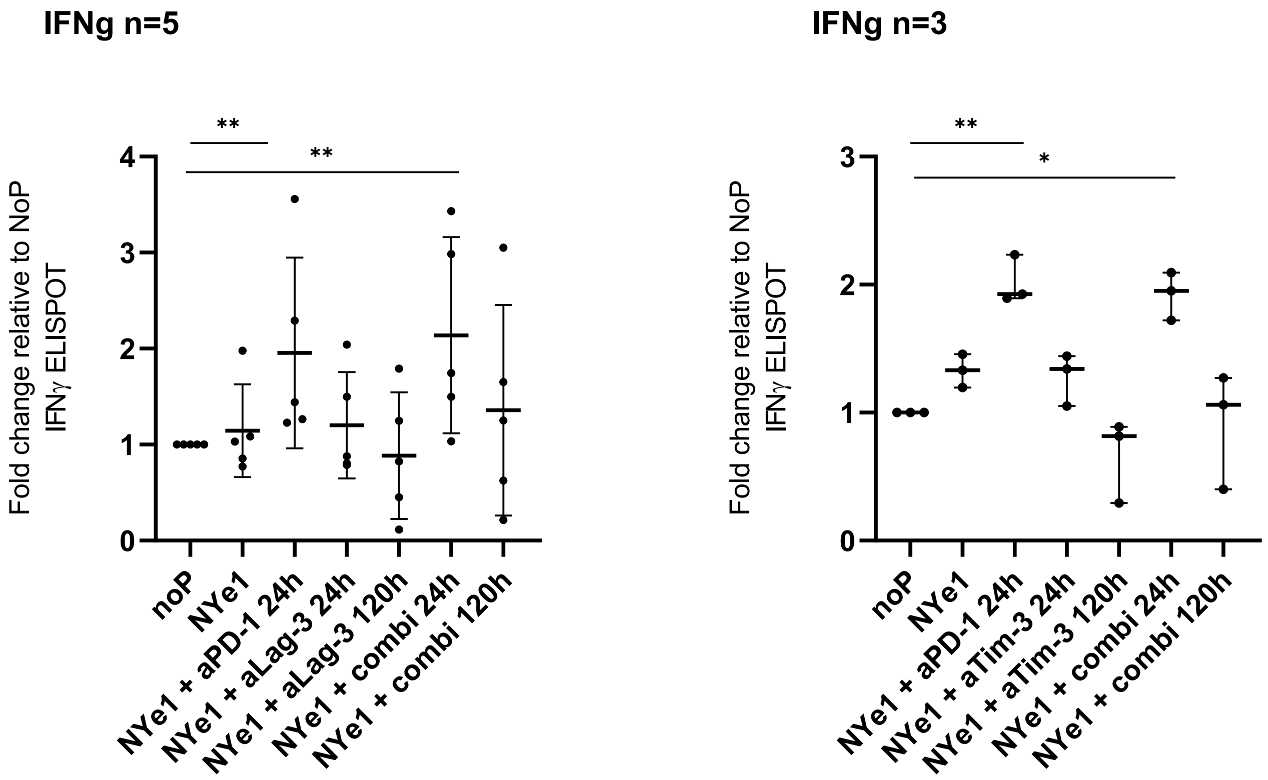

Figure S2 analyses of the addition of immune checkpoints to MLPC at different time points.

Supplementary Figure S3

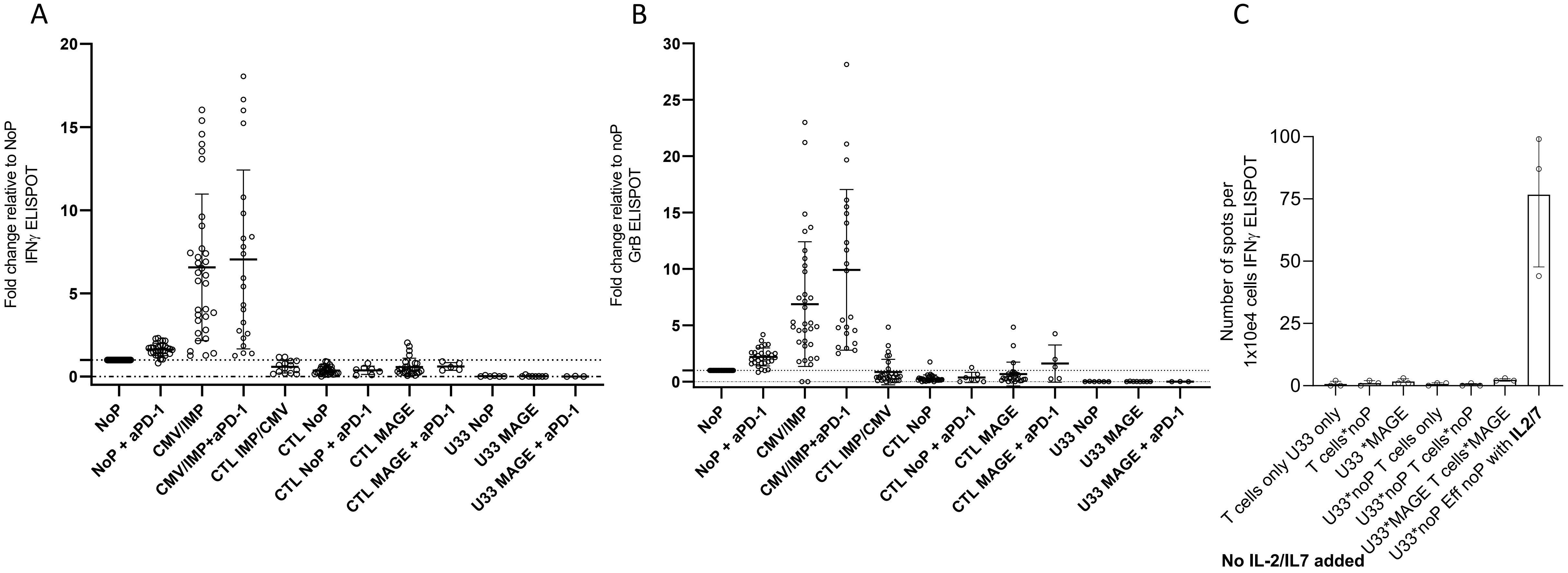

Figure S3 (A, B) controls, showing the relative change compared to untreated immune cells co-cultured with UT-SCC-33 as APC (No Peptide, NoP).

The CTL- and UT-SCC-33 groups showed lower immune stimulation than NoP. The positive controls CMV/IMP were significantly higher than all other values.

All values were significant, when the positive controls were compared to all other values (significance bars not shown due to space limitations).

Figure S3 (C) ELISPOT results by no addition compared to addition of IL-2/IL-7.
